# Supplementary material for: Edge curvature drives endoplasmic reticulum reorganization and dictates epithelial migration mode
Source: Nat Cell Biol. 2025 Aug 18;27(10):1660–75. doi: 10.1038/s41556-025-01729-3 (PMC12527913; doi:10.1038/s41556-025-01729-3)
Supplement: Supplementary file 1 — Supplementary Tables 1 and 2, Note 1 (discussion on curvature analysis) and Note 2 (theoretical modelling and references). [file 41556_2025_1729_MOESM1_ESM.pdf]

nature cell biology

Supplementary information https://doi.org/10.1038/s41556-025-01729-3

Edge curvature drives endoplasmic reticulum reorganization and dictates epithelial migration mode

In the format provided by the authors and unedited

A. Supplementary Table 1: Antibodies and Fluorophores

S.no.	Antibody/Fluorophore	Catalogue No.	Dilution/Working conc.
1.	Alexa Fluor 647 Phalloidin	8940 (CST)	1:40
2.	Alexa Fluor 488 Phalloidin	8878S (CST)	1:40
3.	DAPI (4',6-diamidino-2-phenylindole)	D1306 (Invitrogen)	1 µg/ml
4.	Anti-α-tubulin	3873S (CST)	1:200
5.	Anti-LAMP1	Ab24170 (Abcam)	1:500
6.	Anti-GRASP65	MA5-25148 (Thermo Fischer Scientific)	1:200
7.	Anti-CKAP4(Climp63)	16686-1-AP (Proteintech)	1:200 (IF); 1:2000 (WB)
8.	Anti-RRBP1(p180)	A80974 (Allied Scientific products)	1:200
9.	Anti-Nogo B (Rtn4b)	MA5-32763 (Thermo Fischer Scientific)	1:200 (IF); 1:1000 (WB)
10.	Anti-KDEL	420400 (Merck)	1:200
11.	Anti-ZO1	8193S(CST)	1:200
12.	Anti-Paxillin	Ab32084	1:200
13.	Anti-Sec61β	PA3-015 (Thermo Fischer Scientific)	1:200
14.	Anti-GAPDH	97166S (CST)	1:2000 (WB)
14.	Draq5	4084S (CST)	1:1000
15.	Mito Tracker Green	9074S (CST)	200 nM
16.	Anti-rabbit IgG, AlexaFluor 568	A11036 (Invitrogen)	1:500
17.	Anti-rabbit IgG, AlexaFluor 488	A11008 (Invitrogen)	1:500
18.	Anti-mouse IgG, AlexaFluor 488	A11001 (Invitrogen)	1:500
19.	Anti-mouse IgG, AlexaFluor 568	A11031 (Invitrogen)	1:500
20.	SiR-tubulin	CY-SC006 (Cytoskeleton)	200 nM
21.	SiR-Actin	CY-SC006 (Cytoskeleton)	200 nM

B. Supplementary Table 2: Plasmids

S.No.	Plasmid	Source
1.	mApple Sec61β-C1	90993 (Addgene)
2.	paRac1	22027 (Addgene)
3.	GFP Sec61β	121159 (Addgene)
4.	pEGFP-KHC+	Gift from Hanry Yu
5.	pEGFP-KNT+	Gift from Hanry Yu
6.	pEGFP-Vector	Gift from Hanry Yu
7.	EGFP-EB1c	Gift from Anna Akhmanova
8.	EGFP-Vector	Gift from Anna Akhmanova

9.	Rtn4a-GFP	61807 (Addgene)
10.	mCherry-Climp63	136293 (Addgene)
11.	pYFP-Paxillin	50543 (Addgene)
12.	GFP-MAPPER	117721 (Addgene)
13.	ON-TARGETplus Mouse non targeting pool siRNA	Dharmacon
14.	ON-TARGETplus Mouse Rtn4 siRNA	68585 (Dharmacon)
15.	ON-TARGETplus Mouse Ckap4 siRNA	216197 (Dharmacon)
16.	Rtn 2b GFP	186599 (Addgene)
17.	Rtn3S GFP	169035 (Addgene)
18.	Rtn 4b GFP	186952 (Addgene)

Supplementary Note 1. Discussion on Curvature Analysis

We calculated both single-cell curvature and tissue-level curvature to assess how individual cells respond to geometric constraints at the wound edge. The single-cell curvature (k_{cell}) was determined by fitting a circle to the leading edge of each cell and computing its curvature as $k_{cell} = (R_{cell})^{-1}$, where R_{cell} is the radius of the fitted circle. Similarly, the tissue-level curvature (k_{tissue}) was derived by fitting a smooth curvature-spline to the overall wound boundary and computing $k_{tissue} = (R_{tissue})^{-1}$ where R_{tissue} represents the radius of the wound edge at a given point. To account for variability in how much of a cell is directly interacting with the wound edge, we computed the Fraction of Cell Perimeter Exposed to the Wound Edge (P_{exp}) as $P_{exp} = (L_{edge}/L_{total})X100$, where L_{edge} is the length of the cell perimeter in direct contact with the wound and L_{total} is the total perimeter of the cell. The extent of a cell's perimeter exposed to the wound edge is a critical parameter in curvature analysis, as it determines whether the cell is truly experiencing and responding to the local geometric cue. Without sufficient edge exposure, cellular behavior is more likely to be influenced by neighboring cells than by curvature itself, leading to confounding effects in the analysis. In this respect, at first, plotting ER tubule fractions as functions of P_{exp}, we observed that ER tubule fraction decreased with increasing P_{exp} in the concave region and increased with increasing P_{exp} in the convex region (Extended Data Figs. 2a-b). Next, plotting Climp63 fractions as functions of P_{exp}, we observed that Climp63 fraction increased with increasing P_{exp} in the concave region and decreased with increasing P_{exp} in the convex region (Extended Data Figs. 2c–d). These results suggested that the extent of a cell's exposure to the wound edge (P_{exp}) modulates its ability to sense and respond to curvature, with higher exposure reinforcing curvature-specific ER morphologies - tubules at convex and sheets at concave edges. These analyses together underscore the importance of direct geometric input in driving intracellular reorganization.

Next to find a correlation between k_{tissue} and k_{cell}, we plotted them against each other at both curvatures. We observed that the k_{tissue} and k_{cell} had direct correlation for most values at both curvatures with some off data points (Extended Data Figs. 2e-f). We then checked whether these off-data points represented cells with low curvature exposure. To this end, when we included only those cells with $P_{exp} > 20\%$, most of the off-data points got removed (Extended Data Figs. 2g-h). In the cleaned dataset, cell and tissue curvatures emerged to show a stronger one-to-one correspondence (Extended Data Figs. 2g-h) before. Taking this analysis into consideration, for subsequent analyses of our experimental data, we included only those cells with $P_{exp} > 20\%$ to remove the ambiguity in interpreting cell behavior and establish a robust basis for analyzing curvature-dependent responses. This approach ensured that our curvature measurements accurately reflected the behavior of cells at the migration front, providing a robust basis for analyzing curvature-dependent cellular responses. It also confirmed that individual cells sense and respond to the large-scale geometric constraints imposed by the wound edge if they have a certain threshold exposure to the wound edge.

Supplementary Note 2. Theoretical modelling

Actin cortex model. We modelled the actin cortex using a non-linear, strain-rate dependent constitutive law (Extended Data Figs. 7a-b). This model is capable of generating active forces consistent with those seen in actin stress fibres. The model has been successfully applied to simulate cell reorientation[1], and response of individual and monolayer of cells to mechano-chemical stimuli[2]. The curvature-dependent and temporally dynamic behaviour of the actin cortex observed in our experiments further justifies this modelling approach (Extended Data Figs. 7c-d). Following the actin stress fibre growth model[3], the stress in the actin cortex is split into active and passive components. Active stress (σ^{a}) is assumed to be strain-rate dependent,

and passive stress (σ^p) is assumed to be linear elastic. The total stress in the actin cortex (Σ^{ac}) can therefore be written as the sum of active and passive stresses, as given in Eq. (1)

$$\Sigma^{ac} = \sigma^a + \sigma^p \qquad (1)$$

The actin fibres in the cortex are assumed to be uniformly distributed over the domain. The active stress in each of these fibres is assumed to depend on its strain rate ($\dot{\epsilon}$ (ϕ, ω)) as given in Eq. (2).

$$\sigma^a(\phi, \omega) = \eta(\phi, \omega)\sigma_{max}(1 + \frac{k_v\,\dot{\epsilon}(\phi,\omega)}{\sqrt{1+k_v\dot{\epsilon}(\phi,\omega)^2}}) \qquad (2)$$

where ϕ, ω are the standard angular representation in the spherical coordinate system, and $\eta(\phi, \omega)$ indicates the actin fibre concentration at an angle ϕ, ω (Extended Data Fig. 7e). σ_{max} is the maximum stress that the actin fibre can actively generate, and k_v is a constant. Furthermore, the growth of fibre concentration follows an ODE as given in Eq. **Error! Reference source not found.**

$$\dot{\eta}(\phi, \omega) = (1 - \eta(\phi, \omega))Ck_f - \left(1 - \frac{\sigma^a(\phi,\omega)}{\eta(\phi,\omega)\sigma_{max}}\right)\eta(\phi, \omega)k_b \qquad (3)$$

where, C indicates the calcium concentration, which initiates the fibre formation, while k_f and k_b represent the rate of association and dissociation of fibres respectively. Thus, the solution of Eq. (2) and Eq. (3) gives the active stress of a stress fibre present at an angle (ϕ, ω).

The fibre in the spherical coordinates can be converted to Cartesian coordinate system in 3D using a unit vector **m** along the fibre direction (ϕ, ω), written as,

$$\boldsymbol{m} = sin(\omega)\,cos(\phi)\,\boldsymbol{X_1} + sin(\omega)\,sin(\phi)\,\boldsymbol{X_2} + cos(\omega)\,\boldsymbol{X_3} \qquad (4)$$

where $\boldsymbol{X_1}, \boldsymbol{X_2}, \boldsymbol{X_3}$ are the unit vectors in the Cartesian coordinate system (Extended Data Fig. 7e). Thus, the active stress ($\sigma^a(\phi, \omega)$) can be homogenized following Eq. (5) to obtain the components of the active stress tensor.

$$\sigma_{ij}^a = \frac{3}{4\pi}\int_0^{2\pi}\int_0^{\pi}\sigma^a(\omega, \phi)m_i m_j\,sin(\omega)\,d\omega d\phi \qquad (5)$$

where i,j = 1,2,3 represents $\boldsymbol{X_1}, \boldsymbol{X_2}, \boldsymbol{X_3}$ axes of the rectangular coordinate system, and m_i, m_j are the ith and jth components of the unit vector $\boldsymbol{m}$ (Eq. (4)). Further, the integration of Eq. (5) is performed numerically using a trapezoidal rule assuming 20 equally spaced discrete angles in the ϕ, ω domains. The passive stress tensor can be written in the index notation as follows

$$\sigma_{ij}^p = C_{ijkl}^{ac}\varepsilon_{kl} \qquad (6)$$

Where C_{ijkl}^{ac} represents the components of the stiffness tensor of actin cortex, and ε_{kl} the strain. Total stress in the actin cortex can be evaluated following Eq. (1). Thus, depending on the fibre orientation, the magnitude of individual components of the stress tensor varies and determines the direction of force, and hence the deformation.

Mechanical equilibrium. Further, the total stress (Σ) in the system is obtained by adding the corresponding stress components in the actin cortex (Σ^{ac}), cytoplasm (σ_{ij}^{cyto}), and ER (σ_{ij}^{ER}).

We assume cytoplasm and ER to be passive components and for simplicity, we follow a linear elastic formulation as given in Eqs. (7,8)

$$\sigma_{ij}^{cyto} = C_{ijkl}^{cyto}\,\varepsilon_{kl} \quad (7)$$

$$\sigma_{ij}^{ER} = C_{ijkl}^{ER}\,\varepsilon_{kl} \quad (8)$$

The mechanical equilibrium is then solved using the finite element solver Abaqus 2021 [Dassault systèmes, Simulia Corp] with a small strain formulation.

$$\nabla.\Sigma = 0 \quad (9)$$

Solving Eq. (9) results in the generation of stress in the actin cortex and the corresponding deformation of the cell. We assume stress and strain-free initial conditions. Strain (ε) and strain-rate ($\dot{\varepsilon}$) can be further evaluated in Cartesian coordinates, which will be converted into spherical coordinates following Eq. (10). This will be further used to evaluate stress fibre concentration (Eq. (3)) and update the active stress in the actin cortex (Eq. (2)).

$$\dot{\varepsilon}(\phi,\omega) = \varepsilon_{11}K_1^2 + \varepsilon_{22}K_2^2 + \varepsilon_{33}K_3^2 + \varepsilon_{12}K_1K_2 + \varepsilon_{13}K_1K_3 + \varepsilon_{21}K_2K_1 + \varepsilon_{23}K_2K_3 + \varepsilon_{32}K_3K_2 \quad (10)$$

where,

$$K_1 = \sin(\omega)\cos(\phi)$$

$$K_2 = \sin(\omega)\sin(\phi)$$

$$K_3 = \cos(\omega)$$

Thus, applying this model on a cell with negative curvature, with boundary conditions to constrain displacement in all directions on the bottom edge of the cell (side marked in orange with small dashes, Extended Data Fig. 7a (left)), leads to purse-string type forces resulting in free ends moving towards each other (Extended Data Fig. 7a (middle and right)). Further to use the same contractility model to simulate protrusion under positive curvature, we change the direction of the active stress relative to contraction and restrict the protrusion to Y axis by constraining displacements along X and Z axes of side and top edges (sides marked in black with large dashes, Extended Data Fig. 7b (left)). Additionally, we also constrain the displacement in all directions on the bottom edge of the cell (side marked in orange with small dashes, Extended Data Fig. 7b (left)). This leads to cell protruding along Y axis (Extended Data Fig. 7b (middle and right)). Accordingly, we perform simulations by changing the geometry of the cell, ER morphologies and compare the strain energy density (Eq. (11)) between the cases to find out which morphological state of ER is preferred for a given condition, where V is the total volume of the cell.

$$Strain\ Energy\ Density = \frac{1}{V}\frac{1}{2}\int_V \boldsymbol{\Sigma}\colon \boldsymbol{\epsilon}\, dV \quad (11)$$

During the comparison of ER morphologies, we keep the volume of ER the same between the cases to remove any bias that the amount of ER can introduce on the overall stiffness of the cell. The parameters used in the simulations presented in this article is given in Table1. The results presented in this study were obtained after the simulations reached a quasi-steady state.

Table 1 Parameter values used in the simulations

Parameter	Value	Reference
Young's Modulus of Epithelial cell	73 kPa	Nehls *et al.*[4]
Young's Modulus of ER fibre	19.3 MPa	Georgiades *et al.* [5] and Picas *et al.* [6]
Poisson's ratio	0.45	Estimated
Maximum stress in actin fibre (σ_{max})	1 MPa	Keshavanarayana *et al.*[3]
Hill constant (k_v)	0.001 s	Keshavanarayana *et al.*[3]
Rate of association of actin fibres (k_f)	1 /s	Keshavanarayana *et al.*[3]
Rate of dissociation of actin fibres (k_b)	0.1 /s	Keshavanarayana *et al.*[3]
Diameter of ER tubules	0.1 μm	Wang *et al.*[7]

Geometry of cell. The curvature (κ) presented in the computational model is the curvature of the ellipse at the inflexion point of the edge evaluated following

$$\kappa = \frac{ab}{\left(\sqrt{a^2 \sin^2 t + b^2 \cos^2 t}\ \right)^3} \tag{12}$$

where a and b are the axes length of the ellipse and t is the angle at which the curvature is evaluated (Extended Data Fig. 7f). We keep the length of a fixed, and vary b, and evaluate κ (Eq. (12)) at t=0, to study cells with curvatures ranging from 0 to 0.17 1/ μm, and match the in vitro conditions.

Geometry of ER. Our experiments showed the presence of different ER morphologies depending on the curvature effects. Hence, different ER morphologies, perpendicular, sheet, and parallel are considered in this article. The perpendicular ER is considered to have a circular cross-section while sheet and parallel ER are considered to have rectangular cross-sections (Extended Data Fig. 7g). While perpendicular and sheet ERs represent distinct independent morphologies, we assume parallel ER to represent a cluster of parallel tubules.

From a structural mechanics standpoint, columns are effective in resisting axial loading, while beams and plates are effective in resisting bending moments. Hence, during protrusion, where axial forces are dominant (Extended Data Fig. 7b), ERs arranged like a set of columns exhibited lower strain energy density. In contrast, during contraction, where bending moments are dominant (Extended Data Fig. 7a), ERs arranged like a set of beams or as a sheet exhibited lower strain energy density. In addition, for a given cross-sectional area, the flexural rigidity of a rectangular cross-section is higher than that of a circular cross-section (Extended Data Fig. 7h). The ratio of flexural rigidity of a rectangular cross-section with the depth same as the diameter of the circular cross-section is 1.33, resulting in lower stress in a rectangular beam

than a circular beam. Using beams with rectangular cross-section instead of circular cross-section under contraction led to lowered strain energy density (Extended Data Fig. 7i). Hence, in all the simulations in the article, we model parallel and sheet ERs with rectangular cross-sections (Extended Data Fig. 7g (middle and right)), and perpendicular ER with circular cross section (Extended Data Fig. 7g (left)). However, since the dimension of ER is smaller than that of the cell, the difference in strain energy due to varying cross sections is negligibly small.

While the current model can predict the preferred ER morphology dependent on curvature and mechanical forces, we do not model active reorganisation. Additionally, currently, the distribution of ER in the cell is also phenomenological. Hence, our future work will focus on using the microscopy images of ER as input to the mechanical model. Additionally, the effect of reorganisation of other organelles, such as microtubules, on ER also merits a detailed separate work.

Code availability: The code is made available as open source, available to download from the GitHub page : https://github.com/bkprdp/Curvature_Dependent_ER_Morphology

Supplementary references

1. Vigliotti, A., Ronan, W., Baaijens, F.P. & Deshpande, V.S. A thermodynamically motivated model for stress-fiber reorganization. *Biomech Model Mechanobiol* **15**, 761-789 (2016).
2. Pathak, A., Deshpande, V.S., McMeeking, R.M. & Evans, A.G. The simulation of stress fibre and focal adhesion development in cells on patterned substrates. *J R Soc Interface* **5**, 507-524 (2008).
3. Keshavanarayana, P., Ruess, M. & Borst, R.d. A feedback-loop extended stress fiber growth model with focal adhesion formation. *International Journal of Solids and Structures* **128**, 160-173 (2017).
4. Nehls, S., Nöding, H., Karsch, S., Ries, F. & Janshoff, A. Stiffness of MDCK II Cells Depends on Confluency and Cell Size. *Biophys J* **116**, 2204-2211 (2019).
5. Georgiades, P. *et al.* The flexibility and dynamics of the tubules in the endoplasmic reticulum. *Sci Rep* **7**, 16474 (2017).
6. Picas, L., Rico, F. & Scheuring, S. Direct measurement of the mechanical properties of lipid phases in supported bilayers. *Biophys J* **102**, L01-03 (2012).
7. Wang, B., Zhao, Z., Xiong, M., Yan, R. & Xu, K. The endoplasmic reticulum adopts two distinct tubule forms. *Proc Natl Acad Sci U S A* **119**, e2117559119 (2022).
