# Supplementary material for: Edge curvature drives endoplasmic reticulum reorganization and dictates epithelial migration mode
Source: Nat Cell Biol. 2025 Aug 18;27(10):1660–75. doi: 10.1038/s41556-025-01729-3 (PMC12527913; doi:10.1038/s41556-025-01729-3)
Supplement: Supplementary file 2 — Reporting Summary [file 41556_2025_1729_MOESM2_ESM.pdf]

nature portfolio

Corresponding author(s): Tamal Das

Last updated by author(s): Jun 16, 2025

Reporting Summary

Nature Portfolio wishes to improve the reproducibility of the work that we publish. This form provides structure for consistency and transparency in reporting. For further information on Nature Portfolio policies, see our Editorial Policies and the Editorial Policy Checklist.

Statistics

For all statistical analyses, confirm that the following items are present in the figure legend, table legend, main text, or Methods section.

n/a	Confirmed	
☐	☒	The exact sample size (*n*) for each experimental group/condition, given as a discrete number and unit of measurement
☐	☒	A statement on whether measurements were taken from distinct samples or whether the same sample was measured repeatedly
☐	☒	The statistical test(s) used AND whether they are one- or two-sided<br>*Only common tests should be described solely by name; describe more complex techniques in the Methods section.*
☒	☐	A description of all covariates tested
☐	☒	A description of any assumptions or corrections, such as tests of normality and adjustment for multiple comparisons
☐	☒	A full description of the statistical parameters including central tendency (e.g. means) or other basic estimates (e.g. regression coefficient) AND variation (e.g. standard deviation) or associated estimates of uncertainty (e.g. confidence intervals)
☐	☒	For null hypothesis testing, the test statistic (e.g. F, t, r) with confidence intervals, effect sizes, degrees of freedom and P value noted<br>*Give P values as exact values whenever suitable.*
☒	☐	For Bayesian analysis, information on the choice of priors and Markov chain Monte Carlo settings
☒	☐	For hierarchical and complex designs, identification of the appropriate level for tests and full reporting of outcomes
☒	☐	Estimates of effect sizes (e.g. Cohen's *d*, Pearson's *r*), indicating how they were calculated

Our web collection on statistics for biologists contains articles on many of the points above.

Software and code

Policy information about availability of computer code

Data collection	Fluorescence images were captured using a 60X oil objective (PlanApo N 60X oil NA=1.42, Olympus) and 100X oil objective (UPlanSApo, 100X/1.4 oil) both mounted on Olympus IX83 inverted microscope equipped with scanning laser confocal head (Olympus FV3000). Super-resolution images were acquired using 100X oil objective (Apo TIRF, NA=1.49, WD= 0.12) mounted on a nikon inverted research microscope eclipse Ti2 E Yokogawa with CSU - W1 SoRa unit .
Data analysis	Image brightness, contrast adjustments, overlay, directionality and segmentation was performed in FIJI (ImageJ) FIJI 1.53q Java 1.8.0_172 (64-bit) and is described in the methods section. MDE analysis was done using a code written in MATLAB (Mathworks, v R2022b). Shape index was calculated using CellPose 3.0 for segmentation and MATLAB for quantification. Statistical analysis was performed using GaphPadPrism v.9.5.0 and Microsoft Excel. The code for mathematical modeling is available as an open-source download from the GitHub page: https://github.com/bkprdp/Curvature_Dependent_ER_Morphology

For manuscripts utilizing custom algorithms or software that are central to the research but not yet described in published literature, software must be made available to editors and reviewers. We strongly encourage code deposition in a community repository (e.g. GitHub). See the Nature Portfolio guidelines for submitting code & software for further information.

Data

Policy information about availability of data

All manuscripts must include a data availability statement. This statement should provide the following information, where applicable:

- Accession codes, unique identifiers, or web links for publicly available datasets
- A description of any restrictions on data availability
- For clinical datasets or third party data, please ensure that the statement adheres to our policy

Data supporting the findings of this work are available in this study and its Extended data figures and the Supplementary Information. Source data are provided with this paper. All other data supporting the findings of this study are available from the corresponding author on reasonable request. The time lapse imaging data (otherwise available as supplementary video files) can only be made available upon request due to the large file sizes and associated storage limitations.

Research involving human participants, their data, or biological material

Policy information about studies with human participants or human data. See also policy information about sex, gender (identity/presentation), and sexual orientation and race, ethnicity and racism.

Reporting on sex and gender	Our research foes not cover this field of work.
Reporting on race, ethnicity, or other socially relevant groupings	Our research does not cover this field of work.
Population characteristics	Our research does not cover this field of work.
Recruitment	Our research does not cover this field of work.
Ethics oversight	Our research does not cover this field of work.

Note that full information on the approval of the study protocol must also be provided in the manuscript.

Field-specific reporting

Please select the one below that is the best fit for your research. If you are not sure, read the appropriate sections before making your selection.

☒ Life sciences ☐ Behavioural & social sciences ☐ Ecological, evolutionary & environmental sciences

For a reference copy of the document with all sections, see nature.com/documents/nr-reporting-summary-flat.pdf

Life sciences study design

All studies must disclose on these points even when the disclosure is negative.

Sample size	No statistical methods were used to pre-determine sample size. However, all experiments were designed to ensure robust and reproducible results, with a minimum of 25 cells analyzed per sample in nearly all cases. A slight exception is the photoactivation experiment in Fig. 3g, where the number of cells analyzed was lower due to the technically demanding nature of the assay—each cell required prolonged high-resolution imaging, limiting the throughput. Despite this, the observed effects were consistent across replicates. Overall, our sampling strategy is appropriate for single-cell resolution imaging and subcellular structure quantification, where each cell provides a rich and independent dataset. The sample sizes are in line with previous studies in the field (for example, PMIDs: 34912111, 38867038). Exact n values are reported in the figure legends.
Data exclusions	No data was excluded for analysis.
Replication	All experiments with quantifications were repeated atleast thrice. All experiments with display representations were repeated at least thrice. All attempts at replication were successful.
Randomization	Culture dishes consisted of 80-100 stamp wound with convex and concave curvatures. Images were captured randomly amongst these at different curvature margins. No sub-sampling was done. Cells were measured randomly under different treatment conditions. Samples were not allocated using randomization, as this study involved observational analysis of epithelial cells under defined experimental conditions. All groups were subjected to identical culture, treatment, and imaging protocols to control for potential covariates, ensuring comparability across conditions.
Blinding	Blinding was considered not to be necessary as the effects observed were clear. The module to segment ER on the trainable Weka segmentation was kept the same for all images. Data collection and analysis were not performed blind to the conditions of the experiments.

Reporting for specific materials, systems and methods

We require information from authors about some types of materials, experimental systems and methods used in many studies. Here, indicate whether each material, system or method listed is relevant to your study. If you are not sure if a list item applies to your research, read the appropriate section before selecting a response.

Materials & experimental systems

n/a	Involved in the study
☐	☒ Antibodies
☐	☒ Eukaryotic cell lines
☒	☐ Palaeontology and archaeology
☐	☒ Animals and other organisms
☒	☐ Clinical data
☒	☐ Dual use research of concern
☒	☐ Plants

Methods

n/a	Involved in the study
☒	☐ ChIP-seq
☒	☐ Flow cytometry
☒	☐ MRI-based neuroimaging

Antibodies

Antibodies used	All the antibodies used in the study have been described in supplementary table 1.
Validation	All primary antibodies and fluorophores used in this study were either commercially validated for immunofluorescence (IF) or western blotting (WB), and further internally validated in our laboratory based on expected subcellular localization patterns or band sizes. The commercial information about antibody validation is available on the suppliers homepage. Anti-α-tubulin (CST, 3873S) and anti-LAMP1 (Abcam, Ab24170) were validated for IF and showed characteristic microtubule and lysosomal localization, respectively. Anti-GRASP65 (Thermo Fisher Scientific, MA5-25148) was validated for IF and showed expected Golgi localization. Anti-CKAP4 (Climp63) (Proteintech, 16686-1-AP) and anti-Nogo B (Rtn4b) (Thermo Fisher Scientific, MA5-32763) were validated for both IF and WB; both showed appropriate ER-like distribution and single bands at expected sizes. Anti-RRBP1 (p180) (Allied Scientific Products, A80974), anti-KDEL (Merck, 420400), and anti-Sec61β (Thermo Fisher Scientific, PA3-015) were all validated for IF and showed consistent ER localization. Anti-ZO1 (CST, 8193S) and anti-Paxillin (Abcam, Ab32084) were validated for IF and localized specifically to tight junctions and focal adhesions, respectively. Anti-GAPDH (CST, 97166S), used for WB, showed a single band at the expected molecular weight. All antibody dilutions and experimental conditions are detailed in the supplementary table and respective figure legends.

Eukaryotic cell lines

Policy information about cell lines and Sex and Gender in Research

Cell line source(s)	MDCK-wild type cell line was a gift from Yasuyuki Fujita. These cells were originally sourced from The European Collection of Authenticated Cell Cultures (ECACC, 85011435). EpH4-Ev was obtained from ATCC (CRL-3063).
Authentication	Original cell lines MDCK-Wild type and EpH4-Ev were authenticated at the source.
Mycoplasma contamination	Cell lines used in the study was free of mycoplasma contamination
Commonly misidentified lines (See ICLAC register)	No commonly misidentified cell lines were used.

Animals and other research organisms

Policy information about studies involving animals; ARRIVE guidelines recommended for reporting animal research, and Sex and Gender in Research

Laboratory animals	Species- Mus musculus, Strain- C57/6J, age- Adult females- 8-12 weeks old, Embryos - E13.5-E16.5. All mice were housed in specific and opportunistic pathogen free conditions at an ambient temperature of 19-23 degree C and humidity of 40-60% with a 12:12 hour light dark cycle prior to use.
Wild animals	This study did not involve wild animals
Reporting on sex	The results are independent of the sex of the animals. The development and physiology of the embryonic epidermal tissue is not known to be different in different sexes, therefore sex of the animal was considered a parameter in the study.
Field-collected samples	Study did not involve samples collected from the field.
Ethics oversight	Protocol was approved by the Institutional Animal Ethics committee (IAEC), TIFR Hyderabad on behalf of Committee for the Purpose of Control and Supervision of experiments on Animals (CPCSEA), India

Note that full information on the approval of the study protocol must also be provided in the manuscript.

Plants

Seed stocks	Our research does not cover this field of work
Novel plant genotypes	Our Research does not cover this field of work
Authentication	Our research does not cover this field of work.
