# Supplementary figures and images for: Edge curvature drives endoplasmic reticulum reorganization and dictates epithelial migration mode
Source: Nat Cell Biol. 2025 Aug 18;27(10):1660–75. doi: 10.1038/s41556-025-01729-3 (PMC12527913; doi:10.1038/s41556-025-01729-3)

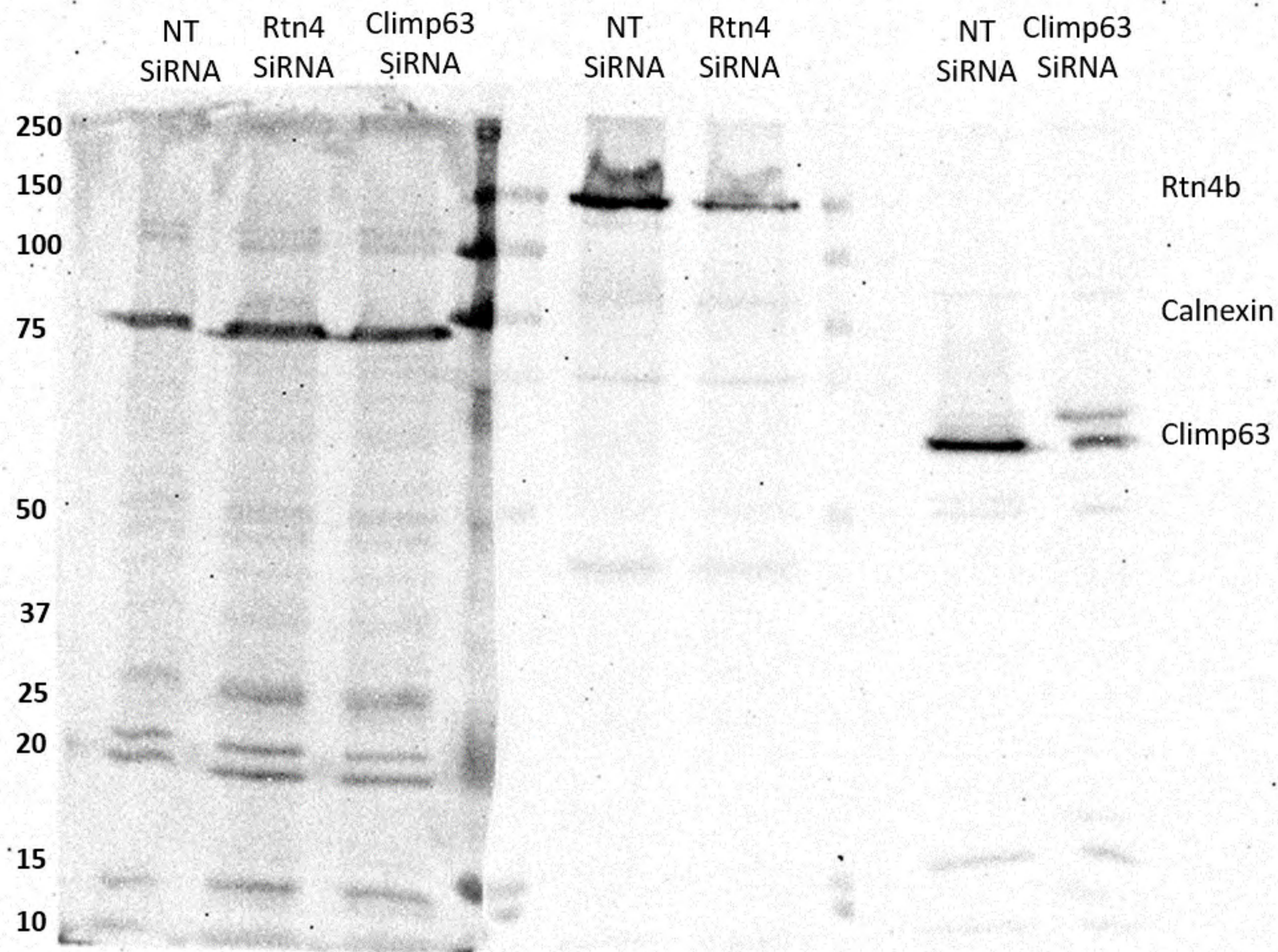

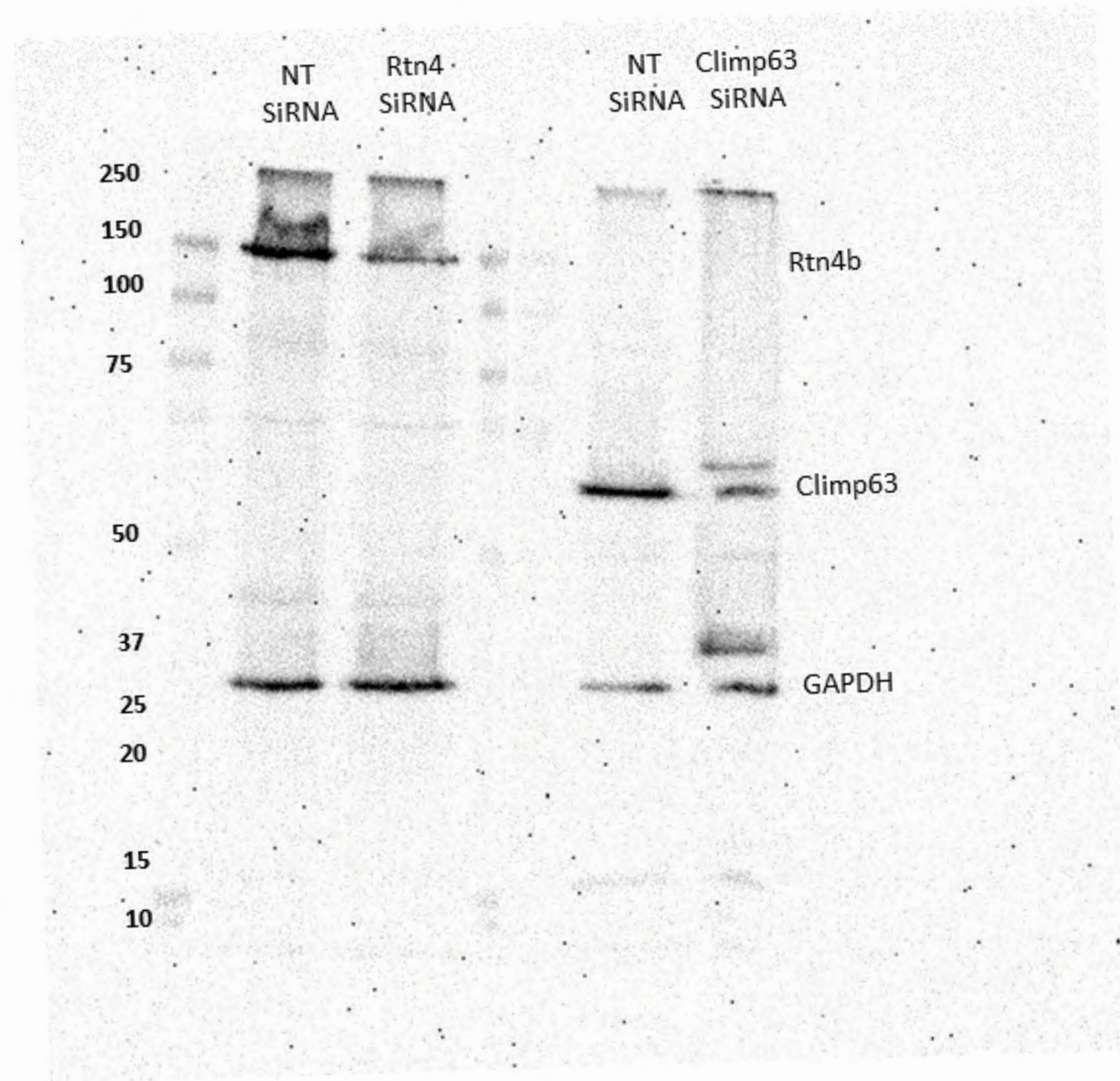

Supplement: Supplementary file 33 — Unprocessed western blots. [file 41556_2025_1729_MOESM33_ESM.pdf]
